# Zinc allocation and re-allocation in rice

**DOI:** 10.3389/fpls.2014.00008

**Published:** 2014-01-27

**Authors:** Tjeerd Jan Stomph, Wen Jiang, Peter E. L. Van Der Putten, Paul C. Struik

**Affiliations:** ^1^Department of Plant Sciences, Centre for Crop Systems Analysis, Wageningen UniversityWageningen, Netherlands; ^2^Crop Cultivation and Physiology Group, Shandong Key Laboratory of Dryland Farming Technology, College of Agronomy and Plant Protection, Qingdao Agricultural UniversityQingdao, China

**Keywords:** ^70^Zn, zinc allocation, rice, *Oryza sativa*, stable isotope, re-allocation

## Abstract

**Aims:** Agronomy and breeding actively search for options to enhance cereal grain Zn density. Quantifying internal (re-)allocation of Zn as affected by soil and crop management or genotype is crucial. We present experiments supporting the development of a conceptual model of whole plant Zn allocation and re-allocation in rice.

**Methods:** Two solution culture experiments using ^70^Zn applications at different times during crop development and an experiment on within-grain distribution of Zn are reported. In addition, results from two earlier published experiments are re-analyzed and re-interpreted.

**Results:** A budget analysis showed that plant zinc accumulation during grain filling was larger than zinc allocation to the grains. Isotope data showed that zinc taken up during grain filling was only partly transported directly to the grains and partly allocated to the leaves. Zinc taken up during grain filling and allocated to the leaves replaced zinc re-allocated from leaves to grains. Within the grains, no major transport barrier was observed between vascular tissue and endosperm. At low tissue Zn concentrations, rice plants maintained concentrations of about 20 mg Zn kg^−1^ dry matter in leaf blades and reproductive tissues, but let Zn concentrations in stems, sheath, and roots drop below this level. When plant zinc concentrations increased, Zn levels in leaf blades and reproductive tissues only showed a moderate increase while Zn levels in stems, roots, and sheaths increased much more and in that order.

**Conclusions:** In rice, the major barrier to enhanced zinc allocation towards grains is between stem and reproductive tissues. Enhancing root to shoot transfer will not contribute proportionally to grain zinc enhancement.

## Introduction

Until recently, crop research on micronutrients largely focussed on alleviating limitations to crop production under deficiency or toxicity conditions (Marschner, 1995; Welch, 1995; Rengel, 1999; Fageria et al., 2002). To this zinc (Zn) was no exception. The interest in plant research on micronutrients changed when it was realized that human mineral deficiencies might be alleviated—at least partly—through improved mineral concentrations in edible parts of major food crops (Graham and Welch, 1996; Welch and Graham, 1999, 2004). This shift from a purely plant production focus to a food chain (Slingerland et al., 2006, 2009) or food systems (Graham et al., 2007) focus also shifted the attention in cereal crops from mainly uptake during early crop development, when Zn deficiency problems tend to be severest, to research on Zn husbandry during all stages of the crop's life cycle.

Such research on Zn husbandry included an analysis of Zn allocation to the grains and the relative role of Zn taken up at different crop development stages. Early research had established there is a strong potential for re-allocation of Zn from vegetative tissues to filling grains in wheat (*Triticum aestivum* L.) when post-flowering uptake was absent (Pearson and Rengel, 1994), despite a partial sequestration of Zn in vegetative organs. Later studies on wheat showed that when post-flowering uptake was made possible also substantial proportions of grain Zn could be accounted for by post-flowering uptake (Garnett and Graham, 2005; Kutman et al., 2011). In nutrient solution experiments with rice (*Oryza sativa* L.), post-flowering Zn uptake equaled or surpassed grain Zn content at maturity over a wide range of applied Zn levels (Jiang et al., 2008a) and over a range of genotypes (Jiang et al., 2008b). Also Mabesa et al. (2013) observed that post-flowering uptake was larger than total grain zinc content over a range of tested rice genotypes.

In the above-mentioned studies, the potential contribution of post-flowering uptake to grain Zn was assessed on the basis of budget analyses. Such Zn budget analyses, however, do not provide full insight into the relative role of re-allocation vs. the role of direct allocation, or into the role of different plant tissues in providing Zn to the grain through re-allocation. Theoretically all Zn taken up after flowering could be allocated to the major transpiring organs (i.e., the leaves and glumes) replacing there the earlier accumulated Zn which could in turn be re-allocated to the grains. This putative role of transpiration in directing allocation of xylem transported Zn has been postulated before (Pearson et al., 1995; Wolswinkel, 1999), but to the best of our knowledge direct evidence is still lacking in literature. Zn is mobile, both in phloem and xylem (Marschner, 1995; Wolswinkel, 1999; Rengel, 2001). Further insight into whether direct allocation implies xylem to phloem transfer in the stem, rachis or grains, or rather in leaves and glumes, and into whether newly acquired Zn in fact substitutes re-allocated zinc will help in identifying most rate-limiting, underlying physiological processes and thus processes to target in breeding for improved Zn content in harvestable parts.

The aim of the current paper is to contribute to our insight into the most rate-limiting steps at organ level throughout crop development. In a next step it would then be possible to couple the insights in and models of the respective membrane transfer, xylem or phloem transport and cell level processes (Grusak et al., 1999; Palmgren et al., 2008; Waters and Sankaran, 2010) to the tissues where and developmental phases when rate limitations seem most important. In addition, Zn re-allocation implies that once allocated to and incorporated into a tissue some Zn can be released again. However, dead leaf tissue contains some Zn, even under severe plant Zn deficiency (Reuter et al., 1997); therefore, part of the Zn, once allocated to an organ, can be considered as sequestered, while additional Zn that is taken up above this minimum level can still be re-allocated. A quantification of Zn sequestration is essential to understand allocation and re-allocation dynamics throughout crop development.

Targeted studies on (re-)allocation that go beyond budget analyses, have been carried out in rice using labeled zinc, either radio-active (Jiang et al., 2007) or stable (Wu et al., 2010, 2011) isotope forms. Jiang et al. (2007) found that most of the Zn taken up by roots after flowering accumulated in grains while only a small portion of leaf-applied Zn was re-allocated from leaves to grains. Recent work of Mabesa et al. (2013) seems to hint into the same direction although there might be genetic variation worth pursuing. These data do not provide information on the contribution of re-allocation from leaves of Zn that had been taken up through the roots at different stages. The study by Wu et al. (2010) focussed on re-allocation under Zn deprivation during a 3-week period directly following a 30-day application of stable isotope Zn providing valuable insights in the way Zn recently taken up is allocated and subsequently re-allocated at different growth stages. However, these data do not provide insight into the long-term re-allocation of Zn taken up during the vegetative stage or during panicle formation to the filling grains after flowering.

In the current paper we report on two experiments with ^70^Zn to study long-term re-allocation of Zn and to distinguish allocation and re-allocation during grain filling of Zn applied during different growth stages. We also report on an experiment carried out to assess whether the observed bottleneck for endosperm loading of Zn in wheat (Stomph et al., 2011) is also present in rice. Moreover, we re-analyse and re-interpret earlier published data on Zn allocation with the aim to identify possible bottlenecks in Zn transfer between tissues.

## Materials and methods

### Experiment 1

A greenhouse experiment was carried out in the UNIFARM facilities of Wageningen University, Wageningen, the Netherlands, in 2009. Seeds of rice (*Oryza sativa* L.) cv. Qinai-3-hun were surface sterilized with 1% sodium hypochlorite for 5 min followed by 70% ethanol for 1 min, after which they were rinsed with deionized water. Sterilized seeds were sown in quartz sand with standing water. Eight days after sowing visibly viable seedlings were rinsed with deionized water to wash off sand and fixed in polyethylene foam discs that fitted in lids of 20 L containers. Per container 20 plants were grown in a 7 × 7 cm grid. The containers were filled with a half strength Hoagland solution without Zn and Fe. This nutrient solution contained 2.5 mM KNO_3_, 2.5 mM Ca(NO_3_)_2_.4H_2_O, 1 mM MgSO_4_.7H_2_O, 0.5 mM KH_2_PO_4_, 46.3 μM H_3_BO_3_, 9.1 μM MnCl_2_.4H_2_O, 0.6 μM CuSO_4_.5H_2_O, 0.03 μM (NH_4_)_6_Mo_7_O_24_.4H_2_O. To this 2.5 ml Fe-EDDHMA per L nutrient solution was added. This chelate is a stable Fe source between pH 3.5 and pH 12. The stable isotope zinc was obtained as ZnO (99.5% ^70^Zn), this was acidified with H_2_SO_4_ (0.01 M ZnO + 0.0195 M H_2_SO_4_) to form ^70^ZnSO_4_. Per 20-L container 1.54 ml ZnSO_4_ 0.01 M (0.05 mg Zn L^−1^) or 1.43 ml ^70^ZnSO_4_ 0.01 M (0.05 mg Zn L^−1^) was added to account for differences in molecular weight of isotopes. The nutrient solution was replaced only at intermediate harvests, i.e., 7 days after panicle initiation (36 days after transplanting) and at flowering (54 days after transplanting). At both moments plant roots were rinsed twice with deionized water and then placed in deionized water for 2 h after which they were placed in the new nutrient solution. This procedure limited carry-over of nutrients. The temperatures in the climate controlled greenhouse compartment were set at 26°C during the 12 h daylight period and 23°C during the 12 h dark period. Nutrient solutions were aerated continuously.

There were three treatments. While growing conditions and nutrient solutions were identical during the full experimental period the plants received stable isotope ^70^Zn during either the period between transplanting and 7 days after panicle initiation (Treatment 1), or the 18-day period prior to flowering (Treatment 2), or the period between flowering and full maturity (Treatment 3). Plants of Treatment 1 were harvested 7 days after panicle initiation, at flowering and at maturity, plants of Treatment 2 were harvested at flowering and maturity, and plants of Treatment 3 were harvested at maturity only. The experiment was carried out in duplicate in a randomized block design. Per replicate of each harvest by treatment combination one container was available with 20 plants arranged as 4 by 5 plants of which only the central 2 by 3 plants were used for observations on biomass and Zn distribution. The organ Zn concentrations for plants of treatments that were not harvested a week after panicle initiation (Treatments 2 and 3) or at flowering (Treatment 3) were assumed to be identical to those of the plants that received stable isotope Zn, while the abundance of ^70^Zn in those plants were assumed to be the natural abundance (0.6%).

Panicle initiation was monitored on some additional plants grown on a separate container. The containers received deionized water three times per week as needed to keep water levels constant. The harvest a week after panicle initiation (36 days after transplanting) was scheduled when panicle initiation was observed on two consecutive harvested plants, the harvest at flowering was scheduled when three of the six central plants on all containers had at least one flowering panicle (54 days after transplanting), while the harvest at maturity was scheduled 96 days after transplanting.

Roots of harvested plants were rinsed with deionized water and then blotted with tissues to remove adhering water. Plants were then dissected into roots, leaf sheaths, leaf blades and, when present, further into stems, rachis and glumes, and grains. At maturity some newly formed tillers were observed and these were harvested separately. Dissected material was dried at 70°C for 48 h before weighing. Hereafter samples were ground in a stainless steel hammer mill and then analyzed for total Zn and percentage ^70^Zn at the Chemical Biological Laboratory of Wageningen UR-Soil Centre. Samples were microwave destructed using HNO_3_-HF-H_2_O_2_ and subsequently samples were analyzed through Inductively Coupled Plasma Mass Spectrometry (ICP-MS).

### Experiment 2

Using the same cultivation method in 20-L containers as reported above, plants of two accessions, Qinai-3-Hun and 90B290, both tested earlier for grain Zn levels under field conditions in China (Jiang et al., 2008b), were grown in growth cabinets of the UNIFARM facilities of Wageningen University, Wageningen, the Netherlands. The basic plant nutrition, day length and temperature settings were as in Experiment 1, but in this experiment two seedlings were placed per foam. In all containers nutrient solutions were only replaced 28 days after transplanting, at panicle initiation of 90B290 (50 days after transplanting) and again at flowering (64 and 79 days for Qinai-3-hun and 90B290, respectively); the experiment was conducted in two replications. The two cultivars were raised at one of three Zn treatments: (1) a control in which plants were grown at 0.05 mg Zn L^−1^, (2) a high Zn treatment in which plants were grown at 3.00 mg Zn L^−1^ during the first 28 days after transplanting, then placed at 1.00 mg Zn L^−1^ until harvest, and (3) a high Zn treatment in which plants were grown at 3.00 mg Zn L^−1^ during the first 28 days after transplanting, then placed at 2.00 mg Zn L^−1^ until harvest. In all three treatments plants received 99.5% ^70^Zn from flowering onwards. Upon harvesting grains were hulled and then polished using a Pearlest^®^ grain polisher (Kett Electric Laboratory, Tokyo, Japan). The polished rice was analyzed at the Waite Analytical Services laboratory in Adelaide, SA, Australia (Wheal et al., 2011).

### Experiment 3

Plants of cultivar Qinai-3-Hun were grown at two Zn treatments on 165 L containers of 1.48 × 0.86 × 0.13 m. The nutrient solution (same as in Experiment 1) was continuously circulated to avoid local nutrient depletion and to provide aeration. The nutrient solution was replaced 15 days after transplanting, at panicle initiation (34 days after transplanting), and at flowering (54 days after transplanting). In the lids of the containers a total of 250 foams could be placed in a 7 × 7 cm grid, and in each foam 2 plants were placed to create a high plant density from the start avoiding profuse tillering and synchronizing flowering. A selection of plants in each container was tagged at flowering with their individual flowering date for later harvesting for this experiment, remaining plants were used for another experiment. A plant was considered to flower when the anthers from at least 5 florets were protruding. Based on the flowering dates individual panicles were harvested at 7, 14, 21, or 28 days after their flowering and at full maturity (35 days after flowering). For each date four individual panicles were selected among the tagged plants. In total six containers were available. Four containers received a standard Zn level (Treatment 1) and two containers a high Zn level (Treatment 2). The four plants were taken one from each standard Zn container providing four true experimental repeats and two plants were taken from each high Zn container providing four replicated plants, but only two true experimental repeats.

Containers for Treatment 1 received 0.05 mg Zn L^−1^ at the start and when the nutrient solution was replaced. Containers for Treatment 2 received 3.00 mg Zn L^−1^ at the start and at the first nutrient solution replacement, at panicle initiation the nutrient solution was replaced by one with 2.00 mg Zn L^−1^; at flowering the nutrient solution was again brought to 2.00 mg Zn L^−1^.

Upon harvesting the fresh grains were dissected under a binocular microscope (×16) into glumes, pericarp, seed coat, vascular bundle, endosperm, and embryo. At 7 days after flowering 20 grains were thus dissected and tissue samples bulked, at 14 days after flowering and later 10 grains were dissected and tissue samples bulked as grain tissue weights had increased. The separation between embryo, seed coat, and endosperm was rather difficult 7 days after flowering and these tissues were later bulked. At grain maturity dissection failed and only whole grains were analyzed. After dissection tissues were dried at 70°C for 24 h and weighed. Thereafter the samples were sent to the Waite Analytical Services laboratory in Adelaide, SA, Australia for chemical analyses (Wheal et al., 2011).

### Re-analysis of data from the literature

Data from Impa et al. (2013) were taken from the supplementary material presented on the journal website (last consulted 30 October 2013). Data from Wu et al. (2010) were taken from their tables presented in the paper. In both case only averages were used. The data from Jiang et al. (2008a) are our own so were used as raw data and further referred to as Experiment 4. Here we will mainly use data not published in the mentioned paper so we provide the essentials on the methods for a quick insight, full details can be found in the original paper (Jiang et al., 2008a). The experiment was carried out in 2005 in the greenhouses of the Chinese Academy of Agricultural Sciences, Beijing. Fifteen-day old seedlings of aerobic rice cultivars Handao502 and Baxiludao were transplanted into foam disks fitted in the lids of 70-liter containers with 55 seedlings per container. Containers were filled with half strength Hoagland solution (pH 5.6 ± 0.1) without Zn. Following the method used by Hoffland et al. (2000) for P, Zn (as ZnSO_4_) was added every 3 days to the solution on the basis of expected dry matter increase, and seven target plant Zn concentrations for total plant dry matter (10, 15, 25, 50, 100, 150, and 200 mg kg^−1^). Total Zn applied between start and end of the experiment for the seven target levels was: 142, 166, 251, 350, 558, 768, 979 μg plant^−1^. Temperatures were set to 28°C/21°C (day/night) and 1000 μmol m^−2^ s^−1^ light was supplemented when it was cloudy.

Plant samples were dried at 75°C for 48 h and ground with a stainless-steel blade blender to a particle size of 0.25 mm. Dried ground plant samples (0.50 g) were digested in a bi-acid mixture (HNO_3_: HClO_4_ = 4:1) and Zn was determined by atomic absorption spectroscopy (AAS SPECTRAA-55; Varian Australia, Mulgrave, VIC, Australia) at wavelength 213.9 nm. Zinc analyses were checked with certified Zn values in standard samples obtained from the Wageningen Evaluating Programme for Analytical Laboratories (WEPAL, Wageningen University, the Netherlands).

### Statistical analysis

Provided statistics are based on GENSTAT analyses. In Experiment 2 contrast analyses were used for the three Zn treatments to make single degree of freedom comparisons first between the low and the two high Zn treatments and then between the two high Zn treatments.

## Results

Per plant brown rice dry matter differed among experiments. Values were 3.0, 2.2, and 1.3 g plant^−1^ in Experiments 1–3, respectively. As plant density was twice as high in Experiments 2 and 3 (408 plants m^−2^) as in Experiment 1 (204 plants m^−2^), differences in brown rice dry matter per unit area were smaller: 612, 880, and 510 g m^−2^ in Experiments 1–3, respectively. For comparison brown rice dry matter in Experiment 4 was 1.4 g plant^−1^ and Impa et al. (2013) report brown rice dry weights of 0.2 to 4.5 g plant^−1^ depending on genotype. In Experiments 1–3 the pH of the nutrient solution increased from the original 5.6 to about 7.5 when solutions were replaced.

### Experiment 1

**Figure 1:**
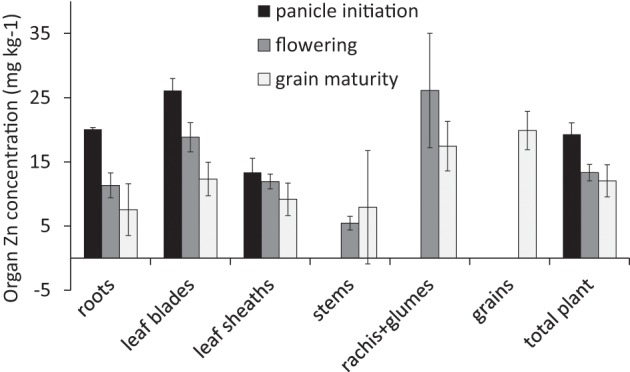
**Zinc concentrations (mg kg^−1^) in the different organs at three harvests averaged across the different ^70^Zn application treatments (Experiment 1)**. Error bars indicate standard errors where *n* = 2, 4, and 6 at panicle initiation, flowering, and grain maturity, respectively.

The Zn level applied in the nutrient solution led to plants with a rather low plant zinc concentration towards maturity (<15 mg kg^−1^, Figure 1). This low overall plant Zn concentration combined reasonable concentrations in leaves during early growth and in grains with very low stem Zn concentrations.

**Figure 2:**
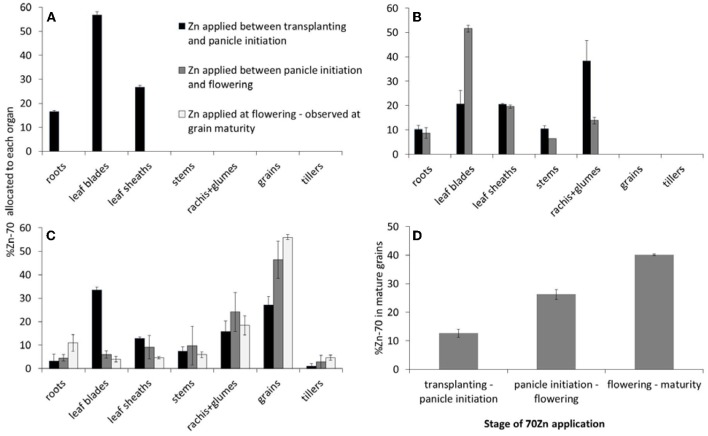
**Allocation of ^70^Zn as observed by destructive sampling at 1 week after panicle initiation (A), at flowering (B), and at grain maturity (C), and as influenced by the period during which the plants received the ^70^Zn (D) (Experiment 2)**. Panel **(D)** shows the calculated percentage of total grain ^70^Zn at grain maturity that can be traced back to ^70^Zn that was applied during the three distinguished development stages. Error bars indicate standard errors (*n* = 2).

One week after panicle initiation the ^70^Zn accumulated since transplanting was allocated to all three distinguished organs, but more than half was observed to be present in the leaf blades (Figure 2A).

At flowering, plants that had received ^70^Zn in the preceding 18 days had allocated roughly half of the accumulated ^70^Zn to their leaf blades, while the remaining organs all received some of the other half, with small differences among organs (Figure 2B, grey bars). Plants that had received ^70^Zn prior to panicle initiation had re-allocated a substantial part of that Zn at flowering. Main net source of ^70^Zn had been the leaf blades (cf. Figures 2A,B), but also roots and leaf sheaths contributed some ^70^Zn to the newly formed stems, rachis, and glumes, whereas the latter two received most of the re-allocated ^70^Zn. Overall, most of the newly acquired Zn went to the leaf blades while these same contributed most to newly formed organs through re-allocation of earlier accumulated Zn.

The ^70^Zn distribution at grain maturity indicated that roughly half of the Zn that had been taken up between flowering and maturity had accumulated in the grains (brown rice), while a further 18% had been accumulated in rachis and glumes, and the remaining was allocated to the other organs, including newly formed tillers (Figure 2C). Of the Zn that had been taken up between panicle initiation and flowering, 45% had been re-allocated to the grains. This re-allocation was mainly at the expense of the leaf blades, but also leaf sheaths contributed (compare grey bars in Figures 2B,C). The ^70^Zn accumulated prior to panicle initiation and that had not been re-allocated to reproductive tissues (rachis and glumes) by the time of flowering (Figure 2B, dark bars) did not contribute during grain filling through re-allocation to rachis, glumes, or grains. Some re-allocation between leaf sheaths and blades seems to have occurred, while roots re-allocated also some of their Zn. The largest change was the re-allocation of Zn from rachis and glumes to grains (compare dark bars in Figures 2B,C).

The observed allocation and re-allocation could also be used to assess how much Zn accumulated at each stage could have minimally contributed to total grain Zn (Figure 2D). For Zn taken up during grain filling this is a direct estimate. For Zn accumulated earlier the estimate is lower than the actual value as the observed contribution of ^70^Zn to total grain Zn does not include the re-allocation of non ^70^Zn that had been accumulated prior to transplanting and that had been allocated to ^70^Zn providing tissues simultaneously with the ^70^Zn. The total reported in Figure 2D only accounts for 80% of grain Zn. Possible reasons for this low percentage are discussed below.

### Experiment 2

The rather high Zn levels in Treatments 2 and 3 did not compromise the white rice production per plant (Table 1). In fact Qinai-3-hun produced slightly better at the higher Zn nutrition levels, while there were no effects on the other cultivar as indicated by single degree of freedom contrast analysis. The Zn concentrations in the polished grains (white rice) were roughly tripled at the higher Zn nutrition level compared to the standard Zn nutrition across the two cultivars. The ^70^Zn enrichment also differed between the high and standard level Zn treatments. With ^70^Zn accounting for about 30% of the grain Zn at the lower Zn level and between 42 and 55% for the high Zn level, the role of re-allocation from vegetative tissues seems to be slightly suppressed when Zn supply levels and plant Zn status are high.

**Table 1:** **Grain weight per plant (brown rice), total Zn concentrations in the polished (white) rice and ^70^Zn (% of total Zn) observed for three treatments imposed on two cultivars (cv) (Experiment 2)**.

**Cultivar**	**Zn treatments**		**Grain weight (g (plant^−1^)**	**Zinc in polished rice (mg kg^−1^)**	**^70^Zn in polished rice (%)**
Qinai-3-hun	1^1^		1.48	14.9	30.1
	2^2^		2.10	51.3	42.0
	3^3^		2.29	52.9	44.8
90B290	1^1^		1.35	14.8	29.6
	2^2^		1.19	38.7	50.3
	3^3^		1.63	48.7	55.2
P	Cv		0.005	ns	0.03
	Zn	1 vs. 2 and 3	0.02	<0.001	<0.001
		2 vs. 3	ns	ns	ns
	Int	Cv × 1 vs. 2 and 3	0.048	ns	ns
		Cv × 2 vs. 3	ns	ns	ns
SED	Cv		–	3.04	2.11
	Zn		–	3.73	2.58
	Int		0.41	–	–

1 0.05 mg Zn L^−1^ nutrient solution;

2 3.00 mg Zn L^−1^ during first 28 days after transplanting followed by 1.00 mg L^−1^ until harvest;

3 3.00 mg Zn L^−1^ during first 28 days after transplanting followed by 2.00 mg L^−1^ until harvest.

### Experiment 3

**Figure 3:**
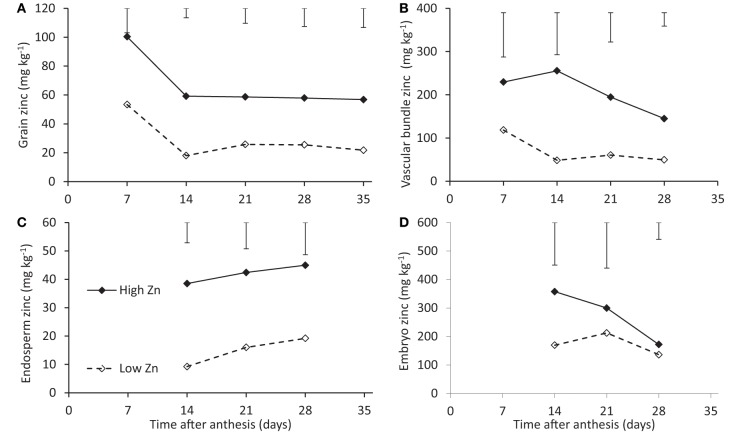
**Zinc concentrations of rice seeds of cv. Qinai-3-hun during grain filling when cultivated on a nutrient solution with either a normal (open symbols) or an extremely high (closed symbols) Zn level (Experiment 3)**. Total grain concentrations **(A)** and concentrations of the vascular bundle **(B)**, endosperm **(C)**, and embryo **(D)** are shown, at 7 days after anthesis no good distinction could be made between embryo and endosperm so tissues were lumped. Note that the scales along the y-axes differ.

Total grain Zn concentration dropped between 7 and 14 days after flowering and remained more or less constant for the remaining time of the grain filling period (Figure 3A). The trends were independent of Zn supply levels, while concentrations were approximately 40 mg kg^−1^ higher at the higher Zn supply level.

The concentrations in the endosperm showed differences between Zn application levels comparable to those for total grain concentrations (Figures 3A,C). Embryo concentrations were an order of magnitude above those of the endosperm and tended to converge for the two Zn application levels (Figure 3D). Vascular bundle concentrations were intermediate between embryo and endosperm and a difference between the two supply levels of about 100 mg kg^−1^ was maintained (Figure 3B). Both embryo and vascular bundle concentrations rather seemed to decrease with time while those in the endosperm slightly increased with time.

### Re-analysis of data from the literature

#### Root to shoot transfer

**Figure 4:**
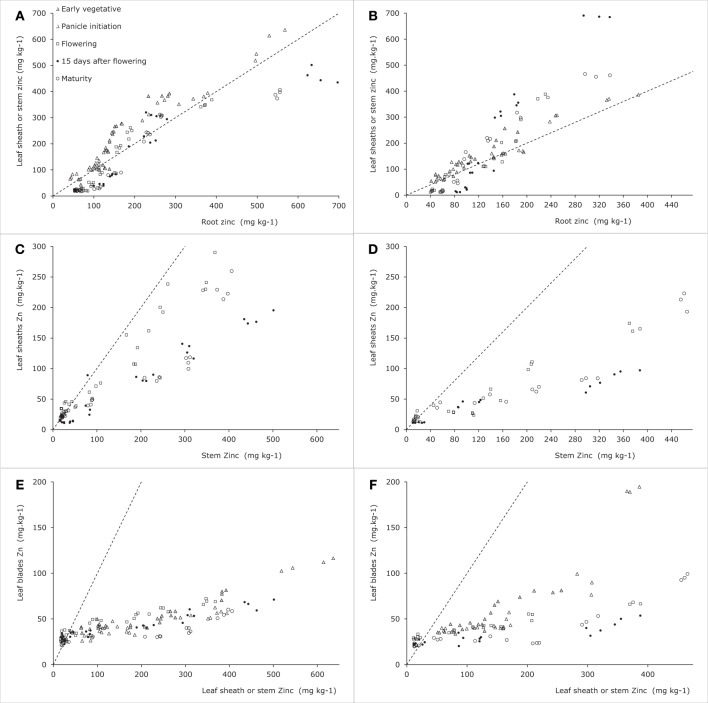
**Zinc concentrations in vegetative plant tissues as indicated along the axes for two rice genotypes tested in Experiment 4 [see also Jiang et al. (2008a)]**. Panels **(A)**, **(C)**, and **(E)** are for Baxiludao, Panels **(B)**, **(D)**, and **(F)** for Handao 502. Data represent individual replications. The filled and open triangles, open squares, black dots, and open circles represent plants during early vegetative growth, panicle initiation, flowering, mid grain filling, and grain maturity respectively, the broken lines indicate the 1:1 line in each panel.

**Figure 5:**
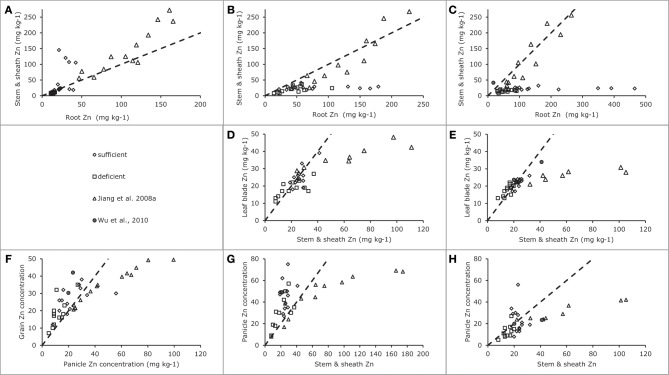
**Zinc concentrations in generative plant tissues as indicated along the axes for data from Impa et al. (2013), Wu et al. (2010) and Experiment 4 [see also Jiang et al. (2008a)]**. Panel **(A)** for plants harvested during the early vegetative stage, Panels **(B)**, **(D)**, and **(G)** for plants harvested at flowering and Panels **(C)**, **(E)**, **(F)**, and **(H)** for plants harvested at grain maturity. Data points from Impa et al. (2013) represent averages per tested genotype grown at either sufficient (diamonds) of deficient (squares) Zn nutrition, data points from Wu et al. (2010) (filled circles) represent averages of two genotypes grown at sufficient Zn nutrition and data points from Jiang et al. (2008a). (Experiment 4) (triangles) represent averages for two genotypes grown at a range of Zn nutrition levels. The broken lines indicate the 1:1 line in each panel.

Data from Experiment 4 indicate that the concentrations in roots tended to be higher than those in leaf sheath (when no stems were present) or in stems at the lower range of tissue Zn concentrations for both tested cultivars (Figures 4A,B). Combining these data at the lower plant tissue concentration range with those from Impa et al. (2013) (Figures 5A–C) shows possible genotypic differences in the root to shoot transfer. While at the early vegetative phase some genotypes showed a much higher concentration in the shoots than in the roots, at later stages the opposite trend was observed as the higher concentrations were only observed in roots rather than in the shoots. In other words root to shoot transfer efficiency seems to differ both among genotypes and between developmental stages.

#### Within-shoot allocation

The allocation between leaf blades and leaf sheaths during the vegetative stage (Figures 4C,D) shows that the two cultivars tested maintained a largely comparable allocation pattern at increasing plant Zn concentrations. The same pattern was observed during reproductive stages between leaf blades and stems. While sheath concentrations in the experiment were not lower than 40–60 ppm during the vegetative stage they dropped well below 20 ppm during the reproductive stages. At these stages plants maintained lower tissue Zn concentrations in the leaf blades than in the leaf sheaths when sheath concentrations were above 20–30 ppm, but maintained leaf blade concentrations consistently above 20 ppm while allowing sheath concentrations to drop as low as 10–15 ppm.

A comparison of leaf sheath and stem concentrations during these reproductive stages shows differences between the two cultivars: Baxiludao maintained higher sheath Zn concentrations than Handao 502 at increasing stem Zn concentrations. Both cultivars, though, had higher stem than sheath Zn concentrations when stem concentrations were above 40–50 ppm, but lower when stem concentrations decreased to 20 ppm.

As Impa et al. (2013) only reported Zn concentrations of leaf blades on the one hand and the combination of sheaths and stem on the other, these have been combined with the same data for Experiment 4 (Figures 5D,E). The data from Impa et al. essentially only report the tissue Zn concentrations where leaf blade and stems plus sheath concentrations were approximately equal with a tendency for leaf blade Zn concentrations to be slightly lower than stem plus sheath concentrations when leaf blade concentrations were above 20 ppm. The data from Experiment 4 overlap with those from Impa et al. (2013) at the lower levels of tissue concentrations and add information for conditions when more Zn would be accumulated.

Once the stem is elongating following panicle initiation the stem plays a central role in the transport between vegetative and reproductive tissues. The different concentrations of organs are therefore plotted against the stem concentrations with the exception of the early leaf blades which are plotted against either the sheath concentration (during vegetative growth) or the stem concentration (during reproductive growth).

#### Generative tissues

**Figure 6:**
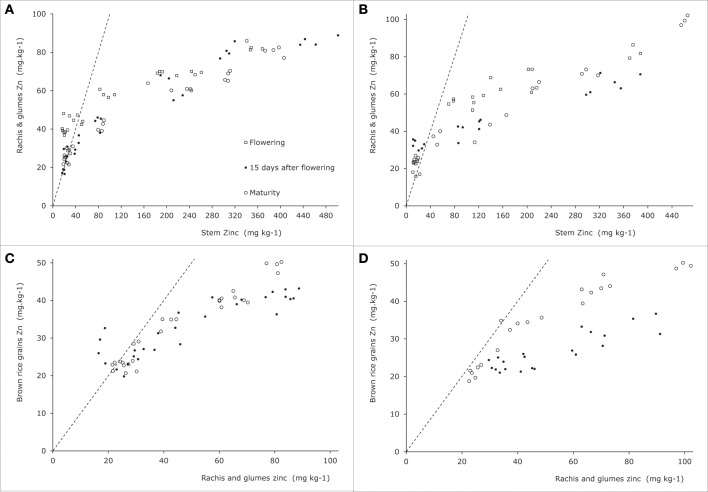
**Zinc concentrations in generative plant tissues as indicated along the y-axes for two rice genotypes tested in Experiment 4 [see also Jiang et al. (2008a)]**. Panels **(A)** and **(C)** are for Baxiludao, Panels **(B)** and **(D)** for Handao 502. Data represent individual replications. The open squares, black dots, and open circles represent plants during flowering, mid grain filling, and grain maturity respectively, the broken lines indicate the 1:1 line in each panel.

As for leaf blades the panicle tissues other than grains (rachis and glumes) had higher concentrations than the stem when the latter had concentrations below 40 ppm, while at higher stem concentrations rachis and glumes maintained concentrations over a much smaller concentration span (Figures 6A,B). The data from Impa et al. (2013) show a different picture at flowering when rachis and glume tissues tended to be higher than stems and sheath concentrations reaching up to 75 ppm at stem concentrations of around 40 ppm while in Experiment 4 such levels were only reached at stem concentrations around 180 ppm (Figure 5G). At grain maturity, panicle tissue Zn concentrations had dropped below that in the stem and sheath generally at stem and sheath concentrations even below 20 ppm (Figure 5H). The data from Experiment 4 seem to be at the lower end of panicle tissue concentrations of the cultivars tested by Impa et al. (2013) especially at flowering (Figure 5G).

During and at the end of grain filling in Experiment 4 (Figures 5C,D) the concentrations in brown rice were always lower than those observed in panicle tissues, while both remained above 20 ppm during these experiments. In the experiments by Impa et al. (2013) lower grain and panicle tissue concentrations were obtained but over the full range of observed concentrations brown rice concentrations were usually higher than panicle tissue concentrations (Figure 5F).

## Discussion

In Experiments 1–3 effects of Zn nutrition were studied over prolonged periods of time using stable isotopes. In contrast to more standard practices the nutrient solution was only replaced a few times in order to avoid regular replacement of the nutrient solution, which would have meant discarding very expensive stable isotopes. The attained grain weights of 510–880 g m^−2^ are reasonable to good rice yields. The un-orthodox nutritional strategy seemed to have resulted in decent plant growth providing results that are comparable to those of other studies. As a consequence of the prolonged plant growth on the same solution, the pH gradually increased from the original 5.6 to about 7.5 by the time the solution was replaced. In soils such high pH values could have led to low availability of Zn to plant uptake. Within this pH range of nutrient solutions, however, Zn availability was hardly reduced. We surmise that Zn availability was not a limiting factor.

The data from Experiment 1 on allocation and re-allocation of Zn in rice suggest that about 40% of seed Zn at grain maturity originated from uptake after flowering, against 26% originating from uptake between panicle initiation and flowering and 12% originating from uptake between transplanting and panicle initiation. The remaining 20% could not be accounted for. This 20% loss is at least partly caused by the root washing method. Root washing might have caused some loss of adhering Zn during transfer from the natural abundance Zn solution to the ^70^Zn containing solution, and vice versa. This effect would have been larger the higher the total root mass was at transfer. So the reported contributions of Zn taken up during grain filling and during the 18 days preceding flowering might have been relatively underestimated compared to the contribution of Zn applied prior to flowering and the Zn applied during the vegetative growth. Despite this imprecision we surmise that, for the accession tested, both allocation of pre- and post-flowering acquired Zn contributed approximately equally to grain Zn. This conclusion could also be drawn from Experiment 2 (Table 1), but this experiment further hints at possible genotypic differences in the relative roles of direct allocation and of re-allocation during grain filling, and at a possible effect of the amounts of Zn that are taken up during grain filling.

These findings contribute to the data from other authors that together provide evidence for genotypic differences in the relative roles of Zn from different sources to grain Zn. Many data at the level of budget analysis indicate that total uptake after flowering at least equals grain allocation (Jiang et al., 2008a,b; Impa et al., 2013; Mabesa et al., 2013). This could be interpreted as an indication that re-allocation would play a very limited role in grain Zn allocation. Yet the data presented here indicate that the dynamics of allocation and re-allocation within the plant are more complex than can be inferred on the basis of budget analyses alone. We have provided arguments that newly acquired Zn may partly replaces earlier acquired Zn in vegetative tissues while the earlier acquired Zn is re-allocated to the seeds. The relevance of such dynamics lies in the possibility to use this information for a more targeted breeding approach. While Jiang et al. (2007) and Wu et al. (2010) found that foliar applied Zn hardly ended up in the grains, recent studies (Phattarakul et al., 2012; Mabesa et al., 2013) suggest that enhancing plant Zn by foliar application does lead to higher grain Zn concentrations. One possible explanation for this apparent discrepancy among studies is that genotypes react differently; an equally plausible explanation based on results presented here is that as leaves are enriched by foliar-applied Zn more Zn from direct uptake is directed towards the grains.

The reported important role for Zn re-allocation from rice stems rather than leaves (Jiang et al., 2008a; Wu et al., 2010) seems to contrast with the results from Experiment 1. Taking a closer look at those data from Jiang et al. (2008a), though, provides evidence that the role of the stem in re-allocation of Zn is a direct function of its role in sequestering Zn that is taken up during pre-grain filling stages in excess of what is needed for optimal functioning of the physiologically most active tissues (e.g., leaf blades). The plant tissue levels in Experiment 1 were such that no extra Zn could be stored in the stem and thus it could not play the same role in providing Zn to the grains that it seemed to have played in the studies by Wu et al. (2010) and Jiang et al. (2008a). The concentration of Zn in the stems observed (10 mg kg^−1^) is of the order indicated by Reuter et al. (1997) to be critical for normal tissue functioning. Most likely the remaining Zn is not available for re-allocation. By combining these results, it can be postulated that plants preferentially re-translocate Zn from the stem unless stem Zn concentration reaches a minimum level below which the remaining Zn is locked in.

The ability to re-allocate Zn from vegetative tissues during grain filling and translocate this Zn to grains will be an important trait especially under field conditions where additional Zn uptake during grain filling might not always be easy. However, an important aspect in breeding for this trait will be to avoid that re-allocation leads to too low levels of Zn in the vegetative tissues that would compromise productivity, especially in leaves. The re-allocation that would make use of senescence enhancing genes to improve grain nutritional quality (e.g., Uauy et al., 2006) seems much less appropriate in environments with a high potential for enhanced production by a prolonged grain-filling phase. Combining the ability to maintain photosynthesis and to accumulate grain Zn would then seem most profitable. In other words a combination of the abilities (1) to accumulate more Zn and maintain higher Zn concentrations during the vegetative stage than is necessary for optimal plant functioning, (2) to re-allocate such Zn to generative tissue while keeping production-relevant tissues at levels that allow optimal functioning, (3) to maintain Zn uptake during grain filling, and (4) to maintain full functioning at relatively low Zn levels in tissues, i.e., a high Zn use efficiency (*in sensu* Hacisalihoglu and Kochian, 2003).

The cultivar studied in Experiment 1 seemed rather effective in re-allocating Zn from its leaves to other plant parts, with a large portion presumably being re-allocated to generative tissues including the grain. This contrasts with observations on re-allocation of foliar-applied labeled Zn in rice. Jiang et al. (2007) found that about 50% of radioactive ^65^Zn applied to the flag leaf was re-allocated, but only around 2% was re-allocated to grains. Wu et al. (2010), working among others with a high Zn density cultivar from IRRI, observed that 18–29% of flag leaf-applied ^68^Zn was re-allocated to other plant tissues and only 4.1–8.9% of flag leaf applied Zn was found in grains. Re-allocation of endogenous Zn might be more effective than re-allocation of leaf-applied Zn in rice. This seems to contrast with what has been observed for foliar-applied labeled Zn in wheat (Erenoglu et al., 2011) and could be an important aspect in the explanation why rice was less responsive to leaf Zn applications than has been reported for wheat (cf. Yilmaz et al., 1997; Phattarakul et al., 2012).

Wu et al. (2011) also showed patterns of accumulation of labeled Zn (^68^Zn) in the rice grain providing further insight into dynamics of direct allocation and re-allocation. Until 15 days after flowering the concentration of directly allocated ^68^Zn increased steeply and also the relative contribution of ^68^Zn increased; thereafter the relative contribution of ^68^Zn remained more or less stable. Direct allocation seems to be of increasing importance at the start of grain filling while later on the relative roles of direct allocation and re-allocation flows remained the same. The role of transpiration in maintaining a xylem influx could be important (cf. Pearson et al., 1996) as transpiration of glumes is bound to decline during late grain filling.

Rice seems to function differently from wheat in the grain transport of Zn during grain filling. For wheat an increase in vascular tissue Zn concentrations was observed towards later stages of grain filling while endosperm concentrations slightly dropped (Stomph et al., 2011). We observed for rice (Experiment 3) rather a decreasing vascular tissue concentration and a slightly increasing trend in the endosperm. This further emphasizes that Zn transport processes differ between plant species even for grasses as closely related as these two cereals (cf. Palmgren et al., 2008; Stomph et al., 2009). The reasons for these differences still need to be elucidated but the role of anatomical differences, like those between rice and wheat grains, should definitely be included in further studies.

**Figure 7:**
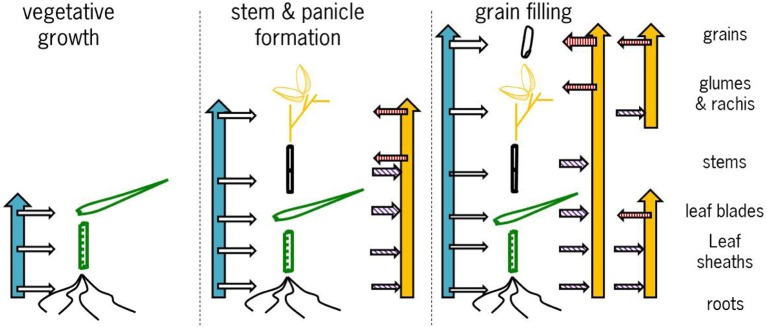
**Graphical representation of the proposed conceptual model of allocation and re-translocation of zinc during the subsequent stages of cereal development**. The blue vertical and the horizontal arrows to the left of each panel indicate uptake and direct allocation to individual tissues, while the yellow vertical arrows to the right of each panel indicate re-allocation flows. The horizontal arrows with purple hatching indicate when tissues are a source of Zn for re-translocation and the horizontal arrows with a red hatching indicate when tissues are sink for Zn from re-translocation. For further explanation see the text.

Combining the data presented here and in the literature the overall picture that emerges is as follows (Figure 7). During the vegetative growth allocation between roots and shoots is directed at maintaining approximately the same concentrations in both tissues, with a slightly higher root concentration. There is some evidence for genotypic differences that may allow breeding for more shoot allocation (Figure 5A). The extent to which there may be some physiological barrier to root-to-shoot transfer varies among genotypes as is evident from the work by Wu et al. (2010) who showed that of the Zn applied during the vegetative stage very different proportions ended up in the shoot (their Table 1). For later stages of plant development the data from Experiment 4 suggest that the relative concentrations maintained in roots and leaf sheath (or sheath and stems when the latter are present) are not very susceptible to the total Zn that is taken up, both increasing proportionally. The data from Impa et al. (2013), though, indicate that there might be genotypes with much greater root-to-shoot transfer barriers as plants develop (Figures 5B,C). For all leaf blades and reproductive tissues there is evidence from Experiment 4 that as overall plant Zn concentrations increase above 40 mg kg^−1^ the Zn concentrations of these tissues increase proportionally much less than Zn concentrations in roots, sheaths and stem (Figures 4–6).

Zinc that is taken up between panicle initiation and flowering (Figure 7, middle panel) is allocated to all developing tissues (data Experiment 1) while also re-allocation takes place from vegetative to reproductive tissues. Depending on the actual total plant Zn uptake this re-allocation between tissues is hidden in a budget analysis as all tissues may increase in both or either Zn content and concentration. The stable isotope studies (Experiment 1; Wu et al., 2010, 2011) clearly show much larger dynamics of allocation and re-allocation of Zn, drivers for which are still not fully understood. For breeding for high grain Zn the exact routes of Zn allocation and re-allocation are not very important, a major point is to enhance the ability to accumulate enough Zn at this stage to allow later re-allocation to the grains.

Comparable dynamics of allocation and re-allocation are also seen during the grain filling stages (Figure 7, right panel). Both direct allocation of newly acquired Zn and re-shuffling of earlier acquired Zn occurred in Experiment 1. The relative Zn concentrations in non-grain panicle tissues and rice stem plus sheaths showed large differences among genotypes (data from Impa et al., 2013, Figures 5G,H) with in general higher concentrations in panicle structures than in sheaths and stem at flowering but equal or lower concentrations in panicle structures at grain maturity. The general decrease in panicle structure Zn concentration between flowering and grain maturity at comparable stem and sheath concentrations hints at a relative transfer barrier between the vegetative and reproductive tissues. For the same set of genotypes grain Zn concentrations were higher than panicle structure concentrations (Figure 5F). Taken together this implies that there is much less barrier for an effective loading of the grain from the panicle structures than for an effective loading of the panicle from the stem and sheath. This loading becomes increasingly less effective when stem and sheath levels are higher as was observed in Experiment 4.

Within the grains, concentrations in the vascular tissues were clearly higher than in the endosperm (Figure 3). When the concentration in the endosperm was corrected for the presence of chemically inactive starch assuming roughly two thirds starch (Stomph et al., 2009), the drop in concentration between vascular tissue and endosperm became negligible and thus does not seem to imply a transport barrier.

In support of breeding the question to answer remains which barriers to tackle in the route from soil to seed. This paper has not addressed the soil to root barriers, which are highly relevant as well. The study by Wissuwa et al. (2008) showed how genotypes that differed substantially at high Zn soil (between 28.2 and 37.7 mg Zn kg^−1^ grain) averaged around 7.8 mg Zn kg^−1^ grain on severely Zn-deficient soils with limited differences between genotypes. Enhancing plant Zn uptake on these Zn deficient soils would at first have increased productivity and thereafter enhanced grain Zn levels. Within the plant there seem to be genotypic and development stage differences in the ease in transfer from root to shoots that could be further studied and linked to quantitative trait loci or markers for marker-assisted breeding. The data presented here clearly indicate that a larger shoot allocation of root Zn will mainly lead to an enhanced Zn concentration in stems and sheath. Priority should therefore arguably be given to the barrier between stem (or stem and sheaths) and panicle tissues. As the transfer between these and the grain seems relatively easy, enhancing stem to panicle transfer is probably going to have a much larger impact on grain Zn concentration. The data from the experiments at the International Rice Research Institute (Impa et al., 2013) suggest some but not a very large genotypic variation among the currently used high Zn lines towards grain maturity but a larger variation at flowering. Further characterization of these dynamics during grain filling could provide insight into possible interesting lines.

## Conclusions

- Both pre- and post-flowering Zn uptake contributes substantially to grain Zn loading when Zn uptake continues after flowering.
- Post-flowering Zn uptake also serves to replace Zn that is remobilized from other organs to the grains, thus maintaining Zn concentrations in tissues like leaf blades.
- The Zn that was taken up prior to panicle initiation was in the stable isotope study remobilized to a lower extent than Zn that was taken up between panicle initiation and flowering.
- In all tissues only part of earlier allocated Zn was remobilized; there seems to be both a pool of Zn that can be re-allocated and Zn that is not available for re-allocation, but is sequestered in vegetative or structural tissues.
- There are transfer bottlenecks between root and shoot and between stem and panicle, alleviating the latter is most effectively going to contribute to grain Zn enhancement.
- The within grain Zn allocation from vascular tissue to endosperm in rice differs substantially from that in wheat.

### Conflict of interest statement

The authors declare that the research was conducted in the absence of any commercial or financial relationships that could be construed as a potential conflict of interest.
